# Examining patterns in medication documentation of trade and generic names in an academic family practice training centre

**DOI:** 10.1186/s12909-017-1015-z

**Published:** 2017-09-22

**Authors:** Alexander Summers, Carly Ruderman, Fok-Han Leung, Morgan Slater

**Affiliations:** Department of Family and Community Medicine, St. Michael’s Hospital, Toronto, ON Canada

**Keywords:** Medication documentation, Prescribing habits, Hidden curriculum, Family medicine, Residency, Generic medications

## Abstract

**Background:**

Studies in the United States have shown that physicians commonly use brand names when documenting medications in an outpatient setting. However, the prevalence of prescribing and documenting brand name medication has not been assessed in a clinical teaching environment. The purpose of this study was to describe the use of generic versus brand names for a select number of pharmaceutical products in clinical documentation in a large, urban academic family practice centre.

**Methods:**

A retrospective chart review of the electronic medical records of the St. Michael’s Hospital Academic Family Health Team (SMHAFHT). Data for twenty commonly prescribed medications were collected from the Cumulative Patient Profile as of August 1, 2014. Each medication name was classified as generic or trade. Associations between documentation patterns and physician characteristics were assessed.

**Results:**

Among 9763 patients prescribed any of the twenty medications of interest, 45% of patient charts contained trade nomenclature exclusively. 32% of charts contained only generic nomenclature, and 23% contained a mix of generic and trade nomenclature. There was large variation in use of generic nomenclature amongst physicians, ranging from 19% to 93%.

**Conclusions:**

Trade names in clinical documentation, which likely reflect prescribing habits, continue to be used abundantly in the academic setting. This may become part of the informal curriculum, potentially facilitating undue bias in trainees. Further study is needed to determine characteristics which influence use of generic or trade nomenclature and the impact of this trend on trainees’ clinical knowledge and decision-making.

## Background

Medications can be referred to by their generic or brand/trade name. The World Health Organization (WHO) recommends the use of a generic, nonproprietary name [1]. In recent years, the use of generic versions of medications has increased [2, 3]; however, generic drugs remain underused [4–7]. Several factors contribute to the persistent use of brand name medications, including skepticism toward generic drugs [8, 9], patients’ lack of awareness of their ability to request generic versions, and pharmaceutical industry marketing that emphasizes brand names [4]. Studies conducted in the United States have shown that physicians most commonly use brand names when documenting medications [10, 11]. Studies have also shown that brand name prescribing is common among residents [12, 13]. A recent study has shown that the nomenclature used by supervising physicians has significant influence on the prescribing patterns of first-year internal medicine residents [12].

The influence of the pharmaceutical industry on clinicians has long been a controversial issue. There has been increasing recognition and concern of bias on a clinician’s knowledge base and clinical decision-making [14, 15]. In the context of medical education, much of the conversation has focused on the influence the pharmaceutical industry can have on medical trainees and how it might impact their choice of treatments for patients [16, 17]. This influence is recognized as a contributor to systemic conflict-of-interests and the transmission of potentially biased information to trainees [18, 19]. In response to this concern, several changes have been made at the undergraduate level, including moving towards using only generic names when discussing pharmaceuticals in lectures and small group sessions [20–22]. However, in postgraduate medical education the majority of training occurs directly within a clinical setting. In this workplace-based education, the informal and hidden aspects of experiential learning [23] have become accepted as a vital component of resident education [24–29]. As such, it is likely that the choices a supervising physician makes in terms of medication nomenclature and prescribing during clinical encounters may impact trainees. However, there is little research to support whether the changes implemented at the undergraduate level regarding nomenclature use have influenced the clinical setting of postgraduate medical education and existing research on patterns and best practices for medication documentation in clinical academic settings is sparse. As such, the objective of this study is to describe the use of generic versus brand names for pharmaceutical products in clinical documentation in a large, urban academic family practice.

## Methods

We conducted a retrospective chart review of the electronic medical records of the St Michael’s Hospital Academic Family Health Team (SMHAFHT). This inter-professional team is based in downtown Toronto, Canada and, at the time of this study, consisted of 60 staff physicians and 40 family medicine residents practicing across five different clinical sites with diverse practice populations. Numerous non-physician health professionals also provide patient care and the site is also a training centre for undergraduate students in medicine, nursing, psychology, pharmacy, dietetics and chiropractic care.

All enrolled patients of the SMHAFHT were eligible for inclusion in the study; patients were excluded if there was no Most Responsible Physician (MRP) assigned to their chart. Within each chart, the Cumulative Patient Profile (CPP) contains up-to-date information relevant to the current health of the patient, including all currently prescribed medications. The medication section of the CPP is populated based on active prescriptions created for the patient or new treatments entered. Health care professionals can write the entire medication name when creating a new prescription/treatment or, after entering just the first few letters, select from a list of corresponding medications options. This search function is dictated primarily by spelling, so entering in ‘lip’ will bring up Lipitor as an option, but will not offer ‘atorvastatin’ as an alternative choice.

Patients who were prescribed any of twenty common medications (Table 1), based on the information in their CPP, were selected for further analysis. These 20 medications were selected because they were representative of a variety of classes of medications (e.g. reflux medications, anti-hypertensives, anti-psychotics, anti-depressants) and, in our clinical experience, commonly prescribed. Different permutations of the generic or trade name were considered synonymous (e.g. esomeprazole, apo-esomeprazole, esomeprazole magnesium, etc.). Over-the-counter medications such as ibuprofen or acetaminophen were not included as there is wide variability in whether practitioners input them into the patient’s CPP compared to prescribed medications. At the time of this study, all medications examined had equivalent generic and brand name options available and no formulary or list of preferred medications existed at this family practice centre.

**Table 1:** List of medications searched in patient CPP

Generic Name	Trade Name	Generic Name	Trade Name
amlodipine	Norvasc	olanzapine	Zyprexa
atorvastatin	Lipitor	pantoprazole	Tecta
citalopram	Celexa	perindopril	Coversyl
clopidogrel	Plavix	quetiapine	Seroquel
desvenlafaxine	Pristiq	ranitidine	Zantac
esomeprazole	Nexium	rosuvastatin	Crestor
gliclazide	Diamicron	sildenafil	Viagra
levothyroxine	Synthroid	tamsulosin	Flomax
losartan	Cozaar	venlafaxine	Effexor
metformin	Glucophage	warfarin	Coumadin

In addition to the medication name, data regarding the patient’s physician and clinical site were extracted from the patient’s medical record. For each physician, the graduation year, through a search of the College of Physicians and Surgeons of Ontario’s (CPSO) website [30] was used to calculate the number of years since graduation. All data were extracted from the electronic medical record (EMR) as of August 1, 2014.

For each of the 20 medications of interest (Table 1), a patient’s chart was classified as including the generic or trade name or both. Descriptive analyses were used to assess the documentation pattern for each medication of interest. The data were also analyzed by physician; we calculated the proportion of medications each physician documented using generic nomenclature. We excluded any data for physicians with less than 6 patients in the study data set from this analysis. Associations between the usage of generic nomenclature and physician characteristics (years since graduation and clinical site) were assessed. All analyses were conducted using SAS version 9.4 (SAS Institute, Cary, NC).

This study received approval from the St. Michael’s Hospital Research Ethics Board.

## Results

As of August 1, 2014, there were 36,372 enrolled patients of the SMHAFHT who had an assigned MRP. Nine thousand, seven hundred and sixty-three (27%) of these patients had one or more of the pre-selected medications documented in the CPP.

**Fig. 1:**
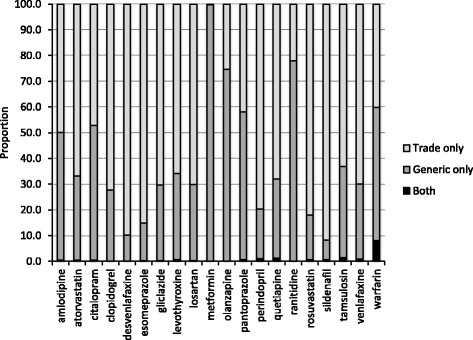
Proportion of patient charts that include only generic names, only trade names, or a combination of both for each specific medication of interest

Only 32% of the 9, 763 patient charts contained generic nomenclature exclusively. Conversely, 45% of patient charts contained only trade nomenclature, and 23% contained a mix of generic and trade nomenclature. There was large variation between the use of generic and trade nomenclature across different medications (Fig. 1). For example, for metformin, the generic name was used in over 99% (1677/1681) of patient charts. Conversely, for sildenafil, the generic name was only used in 7% (41/548) of charts.

**Fig. 2:**
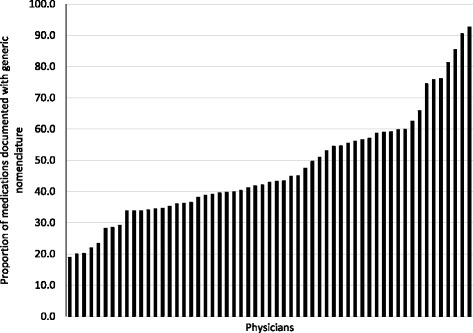
Variation in proportion of medications documented with generic nomenclature by physician

Sixty physicians were included in the study dataset; three had less than six patients with any of these medications in their CPP. These physicians, and their corresponding patients, were excluded from the physician-level analysis. There was large variation between physicians in the use of generic nomenclature, ranging from 19% to over 90% (Fig. 2). There was no association seen across the five sites of the SMHAFHT (*p* = 0.40); however, a slight association was found between the use of generic nomenclature and the number of years since graduation from medical school. The use of generic nomenclature slightly decreased with increasing time since graduation (Pearson correlation coefficient −0.27; *p* = 0.04).

**Fig. 3:**
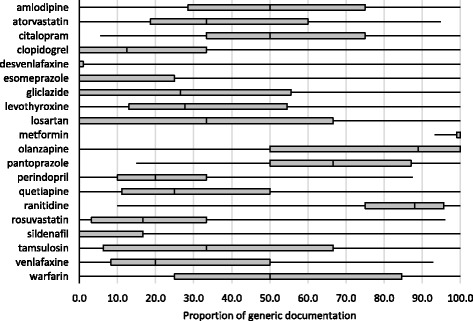
Box plot of the variability in the proportion of generic documentation by physician for each medication of interest

The variation in nomenclature use by physician was markedly different across the twenty medications of interest (Fig. 3). Desvenlafaxine has the lowest usage of generic medication names, compared to metformin with the highest use of generic nomenclature. There is also wide variation in generic use within medication classes; for example, considering two proton pump inhibitors, the median percentage of generic use for esomeprazole is 0% compared to 67% for pantoprazole.

## Discussion

The language used for medications are an important reflection of how physicians practice, especially within an academic setting involved in postgraduate medical education. This is the first study to look at the documentation patterns with respect to prescribing generic versus trade medication names in a Canadian academic family practice unit. Our results show that documentation in the cumulative patient profile significantly favours the use of trade names over generic. This stands in contrast to trends in pre-clinical, didactic medical education across the country, which encourages exclusive use of generic names [20–22]. Based on the functionality of our EMR, where physicians have the choice of selecting either the brand or trade name, it is reasonable to presume that documentation habits reflect prescribing habits. There is some evidence in the literature that suggests that the habitual use of a brand name influences the dispensing of brand-name products, but this primarily refers to the relationship between the physician and pharmacist [7, 31]. We found large variation in documentation patterns across physicians, which was not explained by years since graduation or clinic site. As some practice sites in this family practice unit have recently moved towards removing drug samples and other pharmaceutical influences from the clinical environment, it will be interesting to see whether in time this significantly impacts documentation practices between sites.

There are many possible explanations as to why physicians document using trade names, including variables that affect the rapidity and ease of documentation, such as which name is quicker to write or simpler to pronounce, and patient preference for brand name prescriptions ‘without substitutions’. Physicians themselves may have biases towards brand name products being more effective, despite evidence that the two are bioequivalent, and there is no significant difference between clinical outcomes [32]. Physicians may merely be choosing to use brand names based on familiarity with the brand name (i.e. those medications for which generic products have only recently become available); however, regardless of intent, in a clinical teaching environment the use of trade names becomes part of the informal or hidden curriculum, being absorbed by learners. A number of strategies may mitigate this hidden curriculum. Focused seminars and role-playing interviews designed to address trainees’ knowledge and practices towards the pharmaceutical industry have been shown to affect attitudes and behaviours [33]. Similar continuing medical education (CME) events for physician teachers may encourage reflective practice regarding the implicit biases that can be passed to learners. Explicit institutional policies and standards of professionalism inform the learning environment and can help mitigate these ingrained biases [34]. Lastly, evaluation tools and accreditation guidelines can also be oriented to emphasize the importance of neutrality in medication prescribing.

Moving towards the collective use of generic names for documentation in clinical practice is important for a number of reasons. While patients may be more familiar with brand names, there is value in creating one common, consistent language that is used with patients to decrease patient confusion and improve health literacy. A universal adoption of the WHO’s recommendations to use generic medication names [1] when discussing and dispensing medications to patients, particularly those with poly-pharmacy, may even help avoid medical error. Focusing on the realm of postgraduate medical education, it encourages a neutral learning environment that minimizes bias and industry influence on medical trainees, promoting better educational outcomes. Austad et al. have found a significant association between trainees’ interactions with pharmaceutical promotion and lower odds of selecting an evidence-based prescribing choice [35]. A number of educational interventions aimed at trainees have been described in the literature, such as requiring residents to review their prescribing patterns [36], educational sessions based on patient bills to highlight opportunities for cost-savings [37, 38], and a computerized team-based simulation focused on health care costs [39]. Evidence suggests that focused efforts in training can change the prescribing habits of residents, resulting in the increased use of generic medications [36]. Feedback reports of prescribing performance and one-to-one educational outreach have been shown to be effective at changing prescribing patterns among physicians [40]. With the widespread use of electronic medical records and computerized order entry, a simple solution may be to direct a physician to select a generic name by limiting the medication options to generic nomenclature. These ‘default options’ have been shown to be effective at increasing rates of generic prescribing [41, 42].

The medical profession has long been characterized by a shared language clinicians use to communicate with each other [43]. What is established as the normative vocabulary for this language, and for how we discuss topics from pathology to pharmaceuticals, is reflected and passed on to trainees, as well as to other health care providers, patients, and the general public. Identifying the pervasiveness in trade nomenclature used by clinicians in educational settings is an important component to creating a neutral environment for our learners.

### Limitations

There are a number of limitations to the interpretation of the data collected in this retrospective chart review. Although the use of trade names and generic names are linked to the MRP, it is acknowledged that these are open charts. While the medication portion of the CPP would be predominantly edited by the MRP, however other staff physicians, residents, and pharmacists can make alterations and additions to the chart due to urgent care clinics and resident training. Additionally, this chart review only analyzed twenty medication combinations. These medications were selected because they were commonly prescribed and were representative of a number of classes of medication. However, there are a number of other common medications which were not analyzed in the study for which both generic and trade nomenclature would be used. Finally, the study was limited to one academic family health team; further study is needed to ensure the consistency of our findings across a broader base of academic sites and a wider range of medications.

## Conclusion

Despite increasing efforts to reduce brand name nomenclature in traditional academic settings, the use of trade names in clinical prescribing and documentation is still pervasive. Our study shows that trade names are used abundantly in the medical charts of an academic family health team, with wide variation between physicians. The prevalence of these prescribing and documentation patterns in the educational environment may contribute to the hidden curriculum that informs’ trainees learning and clinical decision making. As the academic medical community reflects on how to best create neutral, impartial environments for learners, the language of our prescribing and documenting should play an important part of the discussion.

## Abbreviations

CPP: cumulative patient profile
CPSO: College of Physicians and Surgeons of Ontario
EMR: electronic medical record
MRP: most responsible physician
SMHAFHT: St. Michael’s Hospital Academic Family Health Team
WHO: World Health Organization
